# Effect of Radiation on the Electrical Properties of PEDOT-Based Nanocomposites

**DOI:** 10.1186/s11671-016-1293-0

**Published:** 2016-02-11

**Authors:** Ivan Karbovnyk, Igor Olenych, Olena Aksimentyeva, Halyna Klym, Orest Dzendzelyuk, Yuri Olenych, Oksana Hrushetska

**Affiliations:** Department of Electronics, Ivan Franko National University of Lviv, 107 Tarnavskogo str., 79017 Lviv, Ukraine; Physical and Colloidal Chemistry Department, Ivan Franko National University of Lviv, 6 Kyrylo and Mefodiy Street, 79005 Lviv, Ukraine; Lviv Polytechnic National University, 12 Bandera str., 79013 Lviv, Ukraine

**Keywords:** Nanocomposites, Electrical conductivity, Polymer, Ionizing radiation, 61.82.Pv, 73.61.Ph, 82.35.Np, 73.63.-b

## Abstract

Systematic evaluation of the influence of radiation on the electrical response of hybrid nanocomposites obtained by adding multi-walled carbon nanotubes into poly(3,4-ethylenedioxythiophene) and poly(styrenesulfonate) host matrix is presented. Variations of resistance and conductivity of nanocomposites depending on the volume fraction of nanotubes in the matrix, ionizing radiation dosage, and temperature are analyzed.

## Background

A possibility of macro-aggregation/compaction of nanotubes in mechanically stable structures with defined geometries opens the way to designing shielding and sensing materials with enhanced performance. The examples of such nanocomposite structures are epoxy resins reinforced with various nanostructures, which have been intensively studied through the last decade [1], or nanotubes incorporated in conducting polymers with conjugated backbone [2, 3]. Specific electrical response of polymers [4, 5] and related nanocomposites [6] makes them good candidates for chemical and biological agent detection applications and, in particular, for ionizing radiation sensing elements [7]. The technology for the design of radiation nanoсomposite sensor is relatively simple and inexpensive. For sensor elements to be efficient, however, the influence of radiation dosage on the conductivity of nanocomposites has to be investigated in details, in order to determine optimal parameters of the element, such as concentration of nanofiller etc.

Particularly interesting example of a conducting polymer is poly(3,4-ethylenedioxythiophene), known as PEDOT [8, 9]. Aqueous dispersion of pre-synthesized PEDOT and poly(styrenesulfonate) (PSS) is often used to create PEDOT-based films. Such films can be deposited on a variety of surfaces, either conductive or dielectric. Depending on a substrate, one can design a flexible or rigid structure. In general, the combination of a good processing ability and electrical properties of the PEDOT:PSS polymer with unique properties of carbon nanotubes [10, 11] looks very promising for designing a nanocomposite with improved characteristics, sensitive to ionizing radiation [12].

Present work reports on PEDOT:PSS/multi-walled carbon nanotube (CNT) hybrid structures and focuses primarily on the effect of ionizing radiation on electrical response of these composites.

## Methods

**Fig. 1:**
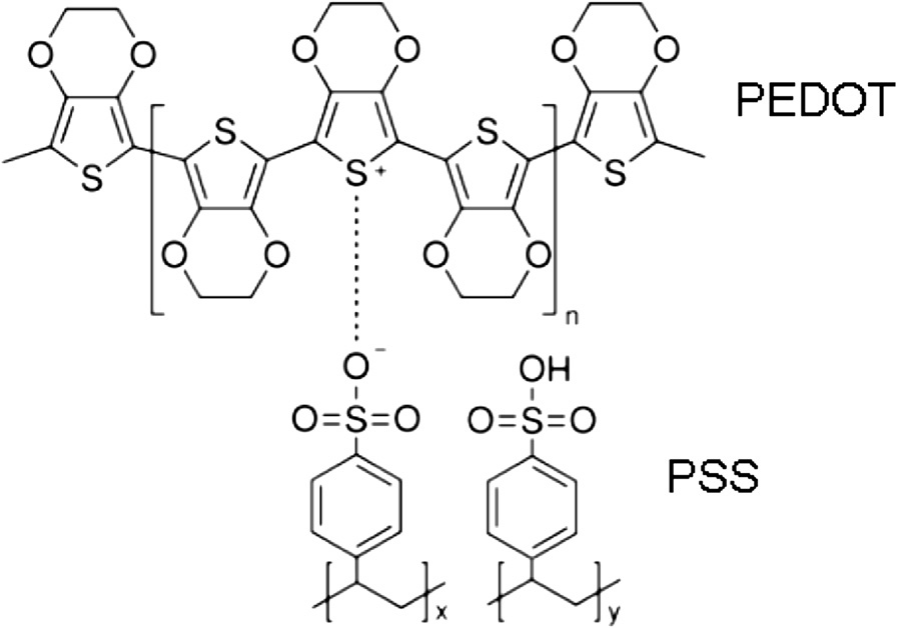
The chemical formula of PEDOT:PSS

PEDOT:PS/CNT structures were prepared by mixing 1.5 % aqueous polymer suspension of PEDOT:PSS with multi-walled CNTs (both components were purchased from Sigma-Aldrich Co, USA). The chemical formula of PEDOT:PSS is shown in Fig. 1.

CNTs added to the polymer had the diameters ranging from 8 to 15 nm and the average length of 30 μm. They were dispersed using an ultrasonic processing in the mixture of nitric and sulfuric acids taken in the 3:1 ratio. The concentration of the CNTs was 0.5 mg per 1 ml. After multiple washing of the CNTs with distilled water, they were mixed with the PEDOT:PSS solution and ultrasonically processed for 8 h. Obtained suspension was deposited onto the optical glass substrate by spin coating and then dried at room temperature during 48 h. Eventually, a monolithic film of the hybrid PEDOT:PSS-CNTs was obtained. The thickness of the film was about 20 μm. Composite PEDOT:PSS films with ~5, ~7.5, and ~10 % CNTs loading were subjected to further experiments. Pure PEDOT:PSS film with no addition of CNTs was measured as a reference.

In order to study electrical properties of hybrid composite films, silver contacts were thermally deposited onto the films surface. The thickness of the contacts was about 0.5 μm, and the distance between them was 5 mm.

Electrical resistivity measurements at room temperature were performed using E7-20 immitance meter (from Calibre, Belarus) in 25 Hz–1 MHz range. Temperature dependencies of AC conductivity were measured with E7-12 instrument operating at 1 MHz. The test signal magnitude was 25 mV.

**Fig. 2:**
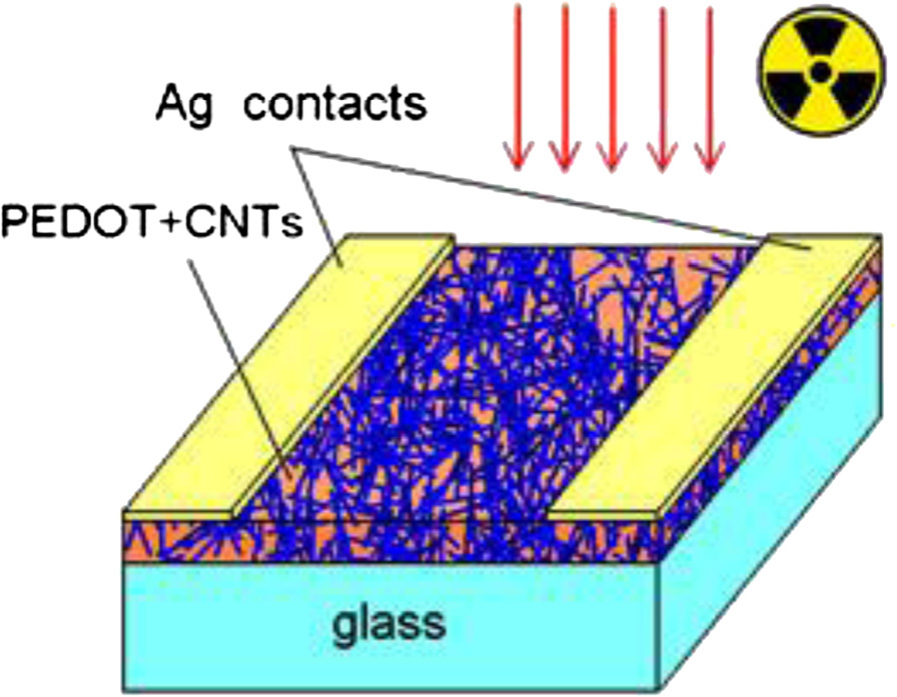
PEDOT:PS/multi-walled CNT structure with contacts. *Arrows* show the incoming radiation from ^226^Ra source

To investigate the influence of the ionizing beta and gamma radiation on the PEDOT:PSS-CNT nanosystems, we have used ^226^Ra isotope with an activity of 0.1 mCi. The decay of ^226^Ra with 3.28 % probability results in the radiation of γ-rays with an average energy of ~0.19 MeV. Produced β-radiation has an average energy of ~0.17 MeV. The irradiation experiment geometry is explained in Fig. 2.

The sample was placed at 0.6 m distance from the radiation source; therefore, there was no need to account for alpha particles, which are typically absorbed already after traveling several centimeters in air. Radiation dosage was estimated considering the exposure time.

## Results and Discussion

**Fig. 3:**
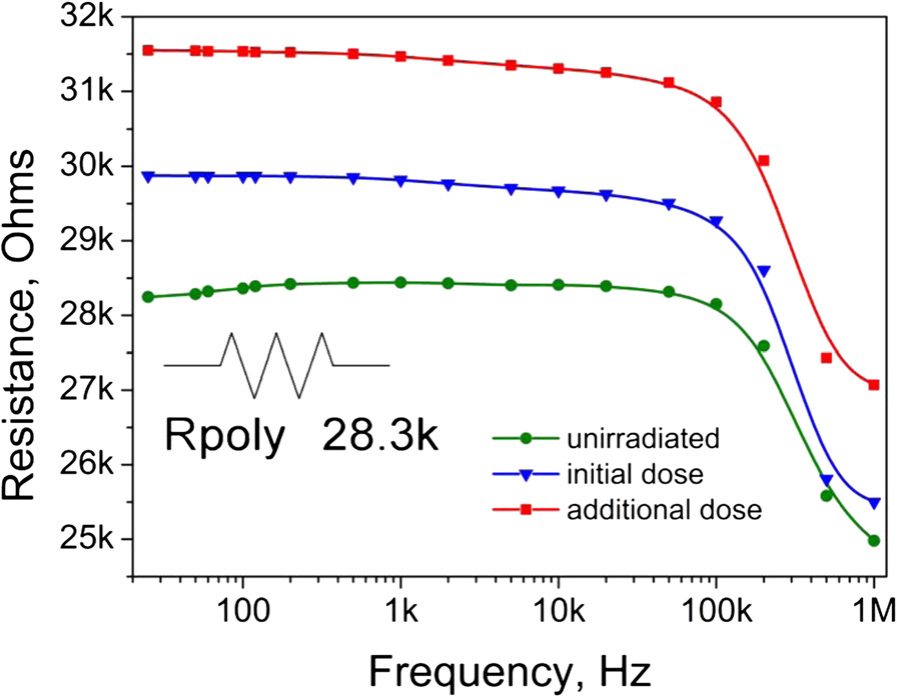
Resistance of pure PEDOT:PSS as a function of frequency

Resistance of the reference PEDOT:PSS sample (with no addition of CNTs) for different doses of absorbed radiation is shown in Fig. 3 as a function of frequency.

Unirradiated sample has resistivity slightly above 28 kOhms in the frequency range from 25 Hz up to 100 kHz (low-frequency range). Resistance drops down to 25 kOhms as frequency increases to 1 MHz. After 30 min of irradiation, resistance in the low-frequency range increases for approximately 2 kOhms and the effect doubles after another 30 min of exposure. Given that below 100 kHz the PEDOT:PSS conductivity is almost frequency independent, one can model the measured system as a simple active resistance *R*_poly_.

**Fig. 4:**
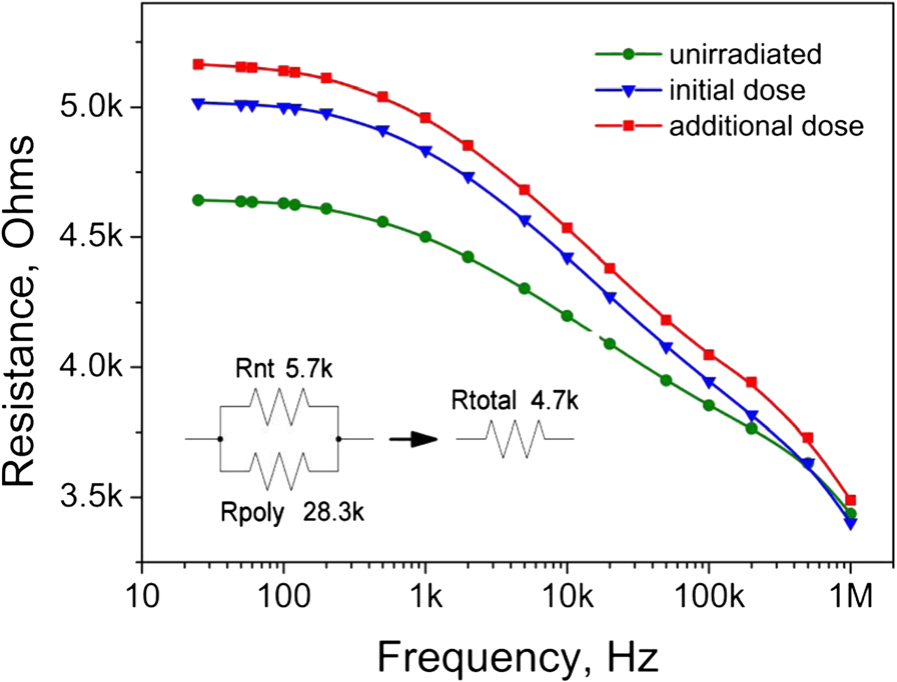
Resistance of pure PEDOT:PSS—5 % CNTs as a function of frequency

There are observable differences in the resistance vs. frequency plots of CNT-reinforced PEDOT:PSS composites (Fig. 4) as compared to the pure PEDOT:PSS samples. The most noticeable effect is that the low-frequency resistance value drops down to 4.7 kOhms for unirradiated sample.

Whereas conductive properties of polymers are somewhat similar to those of inorganic semiconductors [13], the resistance decrease is likely to be due to metallic properties of carbon nanotubes. Assuming CNTs create a concurrent conductive path to that, formed by polymer structure, it is plausible to model the total low-frequency resistance of the PEDOT:PSS-CNT composite as a parallel connection of the aforementioned *R*_poly_ and the nanotube-related resistance *R*_nt_. Knowing this total low-frequency resistance from the experiment (4.7 kOhms) and performing simple calculations for two resistors in parallel, one arrives at the value of 5.7 kOhms for *R*_nt_.

**Fig. 5:**
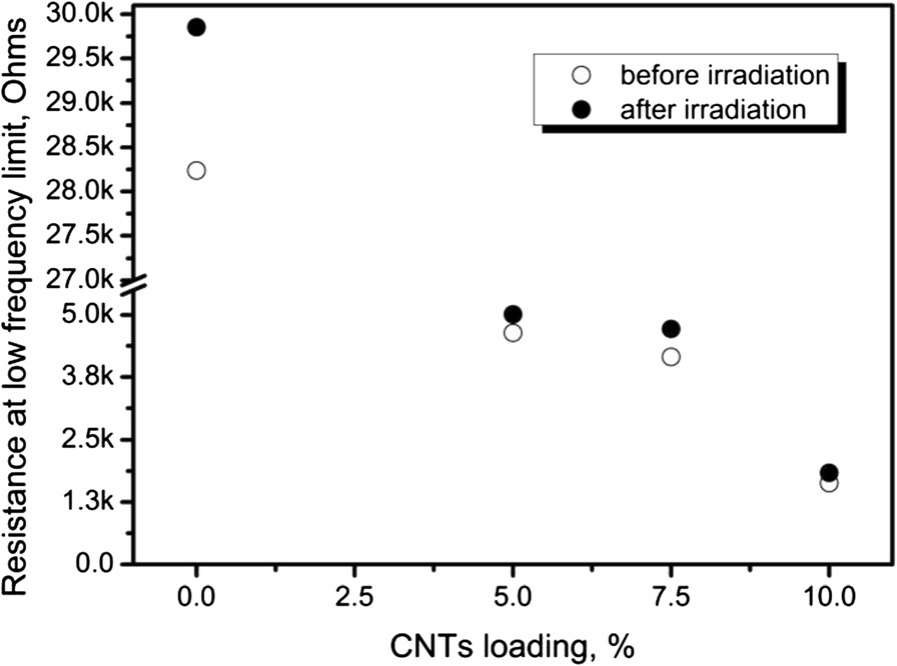
Low-frequency resistance vs. CNT loading

Further increase of CNT loading in the polymer leads to lower resistances of the resulting nanocomposites, as can be seen from Fig. 5.

**Fig. 6:**
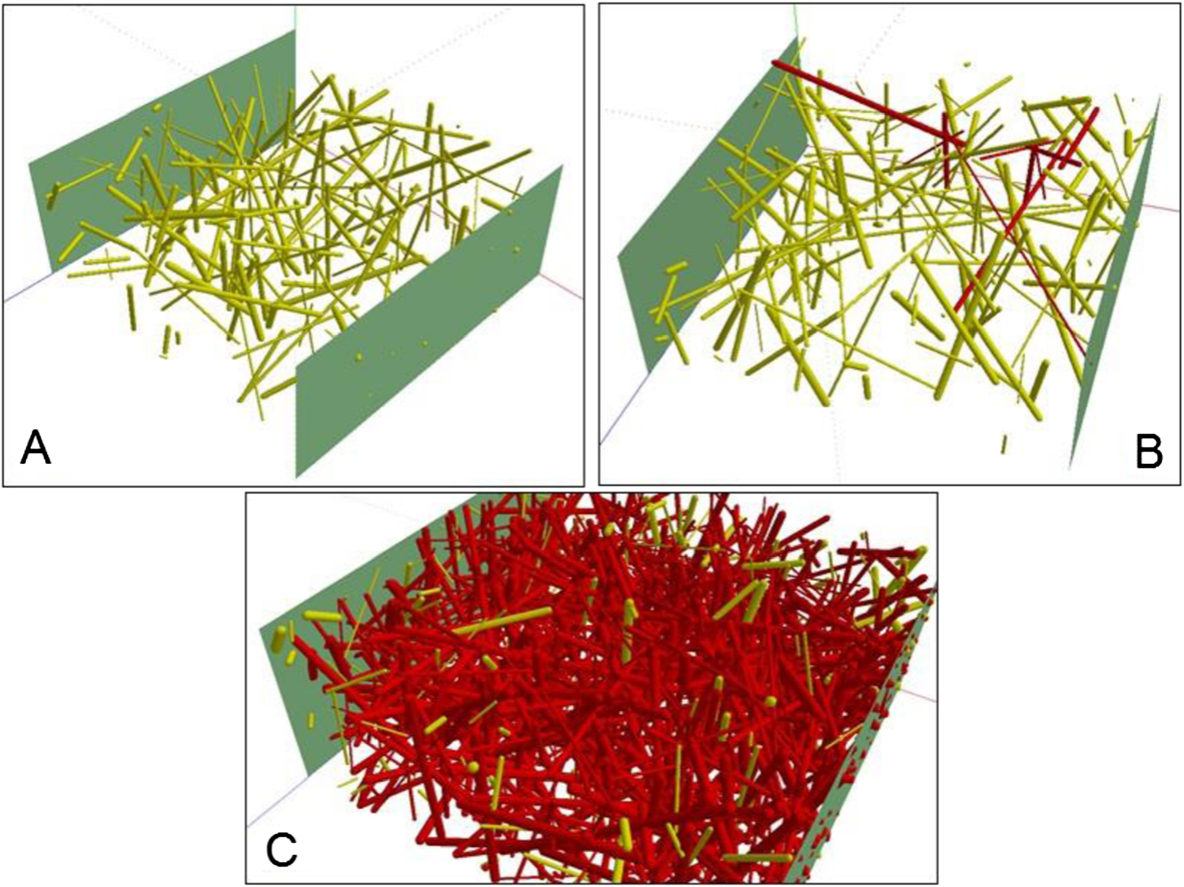
Computer simulation of nanotubes distribution in polymer. **a** no conductive path exists; **b** a single conductive path exists; **c** there exist multiple conductive paths

To support experimental findings, numerical simulations were performed. Polymer matrix reinforced with nanotubes was modeled as a 3D box with “electrodes” attached to its opposite edges, randomly filled with conductive open-cylinder “nanotubes.” Conductive path search algorithm was based on the graph theory. Calculations were run using the same computational resources as in [14]. The results of the calculations allow to visualize the volume distribution of nanotubes inside the host and highlight the conductive paths (see Fig. 6). Calculated minimum volume fraction of nanotubes required for the simulated composite to become electrically conductive, known as the percolation threshold [15], was found to be around 1 %, which is lower than the experimentally observed threshold. The difference can be due to several factors, including model approximations (e.g., limited number of elements in the system as compared to number of CNTs in macro-sized experimental samples) or CNT dispersion conditions during sample preparation.

Figures 4 and 5 also indicate that simultaneous influence of β- and γ-radiation from ^226^Rа source increases the resistance of PEDOT:PSS-CNT composite, as it does in the case of pure polymer (Fig. 3). In case of the composite, such increase should be caused by simultaneous changes in the *R*_nt_ and *R*_poly_. Let us assume the opposite, i.e., that only *R*_poly_ is affected by the radiation. Then, after absorbing the initial dose, the total resistance of the composite would be given by the unchanged *R*_nt_ (5.7 kOhms as determined above) connected in parallel with an increased *R*_poly_ (30 kOhms as can be determined from the blue curve in Fig. 3). This would give a total resistance of 4.8 kOhms, which would be lower than an experimentally observed value (5 kOhms as one can estimate from Fig. 4, blue curve). Thus, the assumption is incorrect, and both radiation-induced changes in *R*_nt_ and *R*_poly_ should be considered.

One of the possible reasons of *R*_poly_ increase could be related to radiation-stimulated concurrent processes of destruction and stitching in the polymer. Polymer chain break due to absorbed ionizing radiation gets in the way of charge transfer through π-electron conjugations, consequently reducing the electrical conductivity of the hybrid system. On the other hand, the appearance of dangling bonds with uncompensated valence electrons allows chain interlocking and stimulates stitching of the polymer. These changes are irreversible, and the degree of transformations depends on the radiation dosage as well as on the polymer structure.

**Fig. 7:**
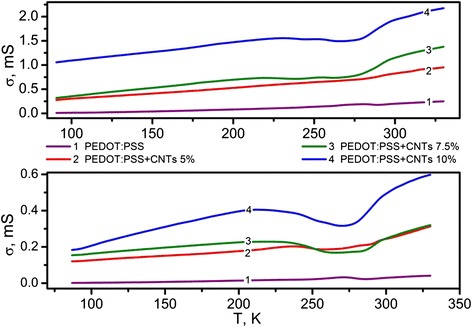
Temperature dependencies of electrical conductivity of PEDOT:PSS-CNT composites at 1 MHz frequency

To have an insight into the processes at the CNTs–polymer interface, we have investigated the temperature dependence of the AC conductivity of the PEDOT:PSS-CNT nanocomposites. The results of the measurements performed in 80–330 K temperature range are depicted in Fig. 7.

Generally, AC conductivity tends to increase upon heating. For lower temperatures, this increase is basically linear. The behavior between 230 and 300 K is more complex. The observed features in this range may be connected with the trapping levels of unequilibrium charge carriers that exist at the interface between polymer and nanotubes. Trapping levels have an influence on the charge transport in the system of π-electron conjugations, which, in turn, is responsible for the electrical properties of conductive polymers [13]. Thus, in PEDOT:PSS-CNT hybrid composites under study, carriers injected from electrodes as well as thermally stimulated ones can contribute to electrical conductivity processes.

Radiation defects that are created in PEDOT:PSS–CNT composites after the exposure result in additional trapping levers being created. Temperature dependencies shown in Fig. 7 confirm the contribution of carriers thermally detrapped from these levels within 230–260 K temperature region.

## Conclusions

Nanocomposites comprising poly(3,4-ethylenedioxythiophene)-poly(styrenesulfonate) and multi-walled carbon nanotubes were synthesized. It was demonstrated that the electrical properties of these composites show high sensitivity to the dosage of β- and γ-radiation. Adding conductive nanotubes allows to decrease resistance while maintaining and even improving radiation sensing properties. Radiation-induced changes occur in the host polymer matrix as well as in nanotubes contributing simultaneously to the total electrical response.
